# Supplementary material for: Small serine recombinases are markers for antiphage defense system discovery
Source: PLoS Biol. 2026 Aug 31;24(8):e3003991. doi: 10.1371/journal.pbio.3003991 (PMC13552941; doi:10.1371/journal.pbio.3003991)
Supplement: Supplementary file 1 [file pbio.3003991.s006.pdf]

Supplemental Table 1 Bacterial/bacteriophage strains, plasmids, and oligonucleotides used in this study.

Category	Relevant Properties	Reference
**Plasmids**		
pLZ12A	P-*bacA* promoter cloned into shuttle vector pLZ12; pSH71 origin; Cm^R	Chatterjee & Johnson *et al.,* 2019
pLZ12A::KAP NTPase / pLZ12A::EF_KAP	pLZ12A constitutively expressing EF_KAP; Cm^R	This Study
pLZ12A::Schlafen	pLZ12A constitutively expressing Schlafen ORF; Cm^R	This Study
pLZ12A::SuperInfection Exclusion	pLZ12A constitutively expressing SuperInfection Exclusion ORF; Cm^R	This Study
pLZ12A::AAA ATPase	pLZ12A constitutively expressing AAA ATPase ORF; Cm^R	This Study
pLZ12A::Phage Head Morphogenesis	pLZ12A constitutively expressing Phage Head Morphogenesis ORF; Cm^R	This Study
pLZ12A::Abi	pLZ12A constitutively expressing Abi ORF; Cm^R	This Study
pLZ12A::Toprim	pLZ12A constitutively expressing Toprim ORF; Cm^R	This Study
pLZ12A::DUF5677	pLZ12A constitutively expressing DUF5677 ORF; Cm^R	This Study
pLZ12A::DUF6056	pLZ12A constitutively expressing DUF6056 ORF; Cm^R	This Study
pLZ12A::EF_KAP D246A,D247A	pLZ12A constitutively expressing EF_KAP D246A,D247A mutant; Cm^R	This Study
pLZ12A::EF_KAP Effector KO	pLZ12A constitutively expressing EF_KAP Effector KO mutant; Cm^R	This Study
pLZ12A::MaG KAP 1	pLZ12A constitutively expressing MaG KAP 1 ORF; Cm^R	This Study
pLZ12A::MaG KAP 2	pLZ12A constitutively expressing MaG KAP 2 ORF; Cm^R	This Study
pLZ12A::MaG KAP 3	pLZ12A constitutively expressing MaG KAP 3 ORF; Cm^R	This Study
**Strains**		
E. faecalis		
OG1RF	Human oral isolate; Rf^R, Fa^R	Bourgogne *et al.,* 2008
OG1RF pLZ12A	OG1RF carrying pLZ12A empty vector	This Study
OG1RF pLZ12A::EF_KAP	OG1RF carrying pLZ12A::EF_KAP	This Study
OG1RF pLZ12A::Schlafen	OG1RF carrying pLZ12A::Schlafen	This Study
OG1RF pLZ12A::SuperInfection Exclusion	OG1RF carrying pLZ12A::SuperInfection Exclusion	This Study
OG1RF pLZ12A::AAA ATPase	OG1RF carrying pLZ12A::AAA ATPase	This Study
OG1RF pLZ12A::Phage Head Morphogenesis	OG1RF carrying pLZ12A::Phage Head Morphogenesis	This Study
OG1RF pLZ12A::Abi	OG1RF carrying pLZ12A::Abi	This Study
OG1RF pLZ12A::Toprim	OG1RF carrying pLZ12A::Toprim	This Study
OG1RF pLZ12A::DUF5677	OG1RF carrying pLZ12A::DUF5677	This Study
OG1RF pLZ12A::DUF6056	OG1RF carrying pLZ12A::DUF6056	This Study
OG1RF pLZ12A::EF_KAP D246A,D247A	OG1RF carrying pLZ12A::EF_KAP D246A,D247A	This Study
OG1RF pLZ12A::EF_KAP Effector KO	OG1RF carrying pLZ12A::EF_KAP Effector KO	This Study
OG1RF pLZ12A::MaG KAP 1	OG1RF carrying pLZ12A::MaG KAP 1	This Study
OG1RF pLZ12A::MaG KAP 2	OG1RF carrying pLZ12A::MaG KAP 2	This Study
OG1RF pLZ12A::MaG KAP 3	OG1RF carrying pLZ12A::MaG KAP 3	This Study
Phages		
G01	*Enterococcus* phage isolated from Ganges River	Sheriff *et al.,* 2024
Npv1	*Enterococcus* phage isolated from sewage	Trotter & Dunny, 1990
VPE25	*Enterococcus* phage isolated from sewage	Duerkop *et al.,* 2016
Phi4	*Enterococcus* phage isolated by the Naval Medical Research Center (NMRC)	Chatterjee & Johnson *et al.,* 2019
Phi17	*Enterococcus* phage isolated by the Naval Medical Research Center (NMRC)	Chatterjee & Johnson *et al.,* 2019
**Sequences**		
ORFs Tested		
KAP NTPase / EF_KAP	Identified in *Enterococcus faecalis* NZ_CP091229.1 (Locus tag: LQ066_14000)	Unpublished
Schlafen	Identified in *Enterococcus lactis* NZ_CP084245.1 (Locus tag: K9N64_RS00170)	Unpublished
SuperInfection Exclusion	Identified in *Enterococcus faecium* NZ_CP175931.1 (Locus tag: ACJXDE_RS13410)	Unpublished
Phage Head Morphogenesis	Identified in *Lactobacillus helveticus* NC_017467.1 (Locus tag: LBHH_RS08930)	Unpublished
Toprim	Identified in *Enterococcus faecalis* NZ_CP091891.1 (Locus tag: L5I18_RS16595)	Unpublished
DUF6056	Identified in *Enterococcus avium* NZ_CP034168.1 (EH197_RS00300)	Unpublished
EF_KAP D246A,D247A	Generated by ATW PCR with EF_KAP D246A, D247A F & R	This Study
EF_KAP Region of Unknown Function KO	Generated by two-piece ATW PCR	This Study
MaG KAP 1	Identified in human fecal microbiome metagenomes	Human Microbiome Project
MaG KAP 2	Identified in human fecal microbiome metagenomes	Human Microbiome Project
MaG KAP 3	Identified in human fecal microbiome metagenomes	Human Microbiome Project
Primers		
EF_KAP D246A, D247A F	GTATTGATCATTGCTGCTTTTGATCG	This Study
EF_KAP D246A, D247A R	CTTTATTTTTTAAAAAGTACGCTGAAATTG	This Study
EF_KAP Region of Unknown Function KO F	TAAGGATCCAGATCTTCCTTCAGGTTATG	This Study
EF_KAP Region of Unknown Function KO R	GTAAGCACCTTCCTGATTTAATTGAGAAATACG	This Study
pLZ12A non-coding region F	NNNNNNCTCGAGGTTTTTTATAAATCTATATTTAAGTAGCTTTATTGTTG	This Study
pLZ12A non-coding region R	NNNNNNCTCGAGGTTGGCGAAAACGTTGGCGATT	This Study
