# Supplementary material for: Small serine recombinases are markers for antiphage defense system discovery
Source: PLoS Biol. 2026 Aug 31;24(8):e3003991. doi: 10.1371/journal.pbio.3003991 (PMC13552941; doi:10.1371/journal.pbio.3003991)
Supplement: Supplementary file 2 [file pbio.3003991.s008.pdf]

Supplemental Table 3 Domains tested in this study from the Bacillota dataset. For a full list of domains identified see Supplemental Table 2.

Domain	InterPro ID	Adjusted P Value	N	ORF Name
KAP family P-loop domain	IPR011646	1.51E-51	18	KAP NTPase / EF_KAP
Schlafen, AlbA_2 domain	IPR007421	2.05E-38	21	Schlafen
Superinfection exclusion protein B	IPR025982	1.46E-16	4	Superinfection Exclusion
Protein of unknown function DUF6056	IPR045691	1.02E-4	3	DUF6056
Phage head morphogenesis domain	IPR006528	7.37E-91	57	Phage Head Morphogenesis
TOPRIM domain	IPR006171	2.57E-305	532	Toprim
